# Micro-Focused Ultrasound Therapy in Patients with Urogenital Atrophy and Vaginal Laxity

**DOI:** 10.3390/jcm11236980

**Published:** 2022-11-26

**Authors:** Piotr Kolczewski, Mateusz Kozłowski, Aneta Cymbaluk-Płoska

**Affiliations:** Department of Reconstructive Surgery and Gynecological Oncology, Pomeranian Medical University in Szczecin, Al. Powstańców Wielkopolskich 72, 70-111 Szczecin, Poland

**Keywords:** micro-focused ultrasound (MFU), micro-focused ultrasound therapy, urogenital atrophy, vaginal laxity, genitourinary syndromes of menopause, aesthetic gynecology

## Abstract

Vaginal laxity (VL) and genitourinary syndromes of menopause (GSM) create physical, psychological, and functional problem for women and their partners. We aimed to evaluate the efficacy and safety of micro-focused ultrasound (MFU) therapy performed twice in the vaginal canal in a patients with VL and GSM. A total of 20 women with GSM and VL were treated with MFU Ultravera by Hironic. The treatment course consisted of two vaginal applications of MFU at an interval of 6 weeks. The clinical effects of the protocol were evaluated using the Vaginal Laxity Questionnaire (VLQ), the Vaginal Health Index (VHI), and the Female Sexual Function Index (FSFI). The overall values of the vaginal laxity evaluation for the total subject population showed a statistically significant improvement between the baseline and the findings at 3 and 6 months after treatment. The effect of therapy was consistent across all domains of FSFI. It peaked at the 6 week follow-up visit (from 26.5 to 32) and plateaued at 12 weeks and 6 months. There was a significant VHI improvement over time, with the greatest and most significant change between the study entry and 21 days after treatment; the VHI score leveled off up to 3 months after the procedures. MFU therapy, performed twice in the vaginal canal, showed promising efficacy and safety profiles, meriting further investigation.

## 1. Introduction

Hypoestrogenism associated with menopause strongly negative affects urinary and vaginal function. In addition, it often leads to GSM (genitourinary syndrome). The term was introduced by the International Society for the Study of Women’s Sexual Health and the North American Menopause Society in 2014 [1]. GSM is related to symptoms such as: atrophic, pale vaginal epithelium with petechiae, dryness, burning, and irritation. In addition, there are the following sexual symptoms: discomfort, pain, and impaired sexual function. This condition, which is also referred to as VVA (vulvovaginal atrophy), is often associated with urinary signs and symptoms: dysuria, urinary incontinence, urgency, and recurrent urinary tract infections [2]. VVA impacts mainly perimenopausal and postmenopausal women, with a frequency from 36% to almost 90%. It has recently been announced that this disorder is already present in premenopausal years, with a frequency of 19% in women aged 40–45 [3,4]. Hypoestrogenism can also occur in other situations, including with the use of gonadotropin-releasing hormone (GnRH) agonist (e.g., in the treatment of endometriosis), surgical menopause, hypothalamic amenorrhea due to excessive exercise, eating disorders, and as a side effect of chemotherapy and radiation therapy, with full clinical symptomatology of VVA [2,3]. Despite this frequent occurrence, VVA is still under-diagnosed and hence under-treated. The majority of women do not debate their symptoms with the gynecologist, for various reasons, often because they think that it is just a normal part of the aging process. Another reason is that they feel ashamed talking about their condition. Often, they are unaware that there are various effective treatments for their problems. No matter what the cause, the absence of diagnosis still remains one of the main issues in the management of this disorder [3,4,5]. Recommendations for GSM treatment depend on the severity of symptoms. For the mild symptomatic GSM, the treatment consists of using long-acting vaginal moisturizers. In the cases of moderate to severe GSM, topical estrogens at low doses have been considered as the standard treatment. Systemic replacement is not always effective in terms of significant improvement of vaginal dryness symptoms. The indicated therapy is then topical treatment with vaginal estrogen (ring or cream) [6,7,8]. There is currently no universal definition for vaginal laxity, or ‘looseness’ [9]. However, it is believed that pregnancy and delivery play a role in the development of this condition. Transvaginal delivery can lead to loss of physical sensation and decreased satisfaction during sexual intercourse. In addition, there is a connection between objective parameters of pelvic floor function in the postpartum period. [10]. It is commonly assumed that transvaginal labor may lead to pelvic floor trauma, especially regarding the levator ani muscle [11]. Both transvaginal labor [12] and levator injury [13] are connected with the increased diameter of the hiatus of the levator ani muscle. An international study of urogynecologists showed that 83% of the 563 respondents reported vaginal laxity as under-reported by the patients. In addition, most of them believed it to be a self-reported bothersome condition that impacts on relationships and sexual function [14,15]. VL is related to younger age, parity, stress urinary incontinence, symptoms of prolapse, and overactive bladder [11,16]. In accordance with research conducted in Western countries, VL has been identified in 24% to 38% of women visiting a urogynecologist [16], and has been noted in 16% of women visiting a plastic surgeon [17]. VL may lead to impaired sexual health. As previous studies have shown, the criteria for sexual distress (Female Sexual Distress Scale, revised score) were met by 46%, and the criteria for female sexual dysfunction (measured by the Female Sexual function Index) were met by 65% of females with VL [17]. Additionally, VL was related to decreased vaginal sensation during intercourse and a generally poorer sexual life [16]. Not surprisingly, it seems that an growing number of females are having vaginoplasty, labiaplasty, and other genital cosmetic procedures [15,18]. A variety of therapies are used to treat VL, and surgery is considered as more effective than physical therapy or Kegel exercises [9]. Surgical procedures, in which vaginal and surrounding tissues are incised, excised, and rearranged, can offer a much better and longer lasting final result. The results of surgical vaginoplasty, however, have to be balanced against the much greater risks involved in any surgery performed in this region. Downtime for recovery is longer, and there are recognized risks associated with scar formation or nerve damage leading to problems with sensation [19,20].

Presently, there are numerous radiofrequency and laser energy-based noninvasive devices targeting the vaginal wall for so-called vaginal rejuvenation, referring to both vaginal laxity and the atrophy of the vaginal epithelium. Thanks to the thermal effect, the atrophic vaginal epithelium becomes thicker, and the concentration of collagen and elastin in the vaginal wall increases. The effectiveness of RF and lasers is supported by many clinical and histological evidences [21,22,23,24,25,26,27,28,29]. However, in 2018, the FDA (Food and Drug Administration), followed by the ACOG and IUGA, launched their critical position requesting more research to prove their safety and effectiveness, providing some recommendation for patients and care providers [19,30]. In response, a group of experts published a consensus based on 20 peer-reviewed publications demonstrating unequivocal effectiveness, as well as minimal short- and long-term complications of RF and lasers in treating GSM [28]. Taking into account their wavelength, CO2 and erbium lasers concentrate all the energy superficially on the epithelium and lamina propria, exerting no deeper action. According to previously published studies [28], radiofrequency (RF) allows for a deeper penetration than lasers; however, a theoretically adequate in-depth thermal effect is still under discussion [31,32]. Many research studies indicated that HIFU (high-intensity focused ultrasound) energy is under consideration to infiltrate tissues in deeper layers than do the standard laser or RF current treatments. Ultrasound waves infiltrate layers and cause vibrations at the beam location. The level of penetration depth is defined by the frequency that characterizes the wave. Thus, shallow frequency waves create focal thermal injury zones (TIZs) across deeper layers. In turn, the formation of lower focal areas of injury is caused by higher frequency waves [33].

A differentiation should be made between the two kinds of ultrasound therapies applied in medical practice: HIFU (high-intensity focused ultrasound) and MFU (micro-focused ultrasound) [34]. HIFU utilizes a high-energy ultrasound beam and is mainly employed for medical purposes, such as non-surgical tumor ablating [34,35]. HIFU also has another application: adipose tissue ablation. For instance, HIFU applied to adipose tissue ablation utilizes 47–59 J/cm^2^ of energy, a frequency of 2 MHz, and a focus depth of 1.1–1.8 cm to ablate subcutaneous fat and accomplish body circumference reduction [36,37,38]. To induce cell disruption and subsequent cell death, the activity of HIFU is both thermal and cavitation. The trauma that results when HIFU is performed on living tissue is the effect of a thermo-mechanical reaction. This involves two separate but integral processes. The first mechanism involves the absorption of ultrasound energy by the tissue. This is followed by the generation of vibrations at the molecular level. The result of this process is the production of heat and a consequent quick increase in temperature in the focal area. Second, the repetitive compressions and rarefactions that exist as ultrasound waves propagate through tissue generate intense shear forces. At the cells level, this tiny shear movement causes heating by friction [39]. Microbubbles, which are created in biological fluids, increase in size until imploding eventually occurs. The forces created by the collapse of the vesicles can lead to cell death through mechanical mechanisms [40]. In contrast, MFU utilizes a much lower energy to treat the skin at the superficial layer level. MFU energy is characterized by the following parameters: 0.4–1.2 J/mm^2^, 4–10 MHz, and a focal depth of 1.5–4.5 mm [41]. To achieve an effect on tissues, MFU depends solely on heat. Although MFU uses lower energy, it is able to bring tissue temperatures to over 60 °C. In doing so, it produces thermal coagulation points, with an area of <1 mm^3^ and a depth of up to 5 mm (in the middle and deep reticular layers of the dermis and subcutis). It should be noted that in the process, the papillary, dermal, and epidermal layers of the skin above them are not destroyed [42,43]. The MFU device was launched in 2009. The goal was to deliver a precisely targeted region of thermal injury at a treatment depth greater than that offered by RF and laser techniques [44]. In addition, to prompt more extensive collagen remodeling, thermal energy is supplied to the superficial dermis and subcutaneous connective tissue [45]. Earlier research pointed out that differences in small pulse durations, in combination with higher frequency transmitters, allow MFUS to generate targeted zones of necrotic coagulation, called TIZs, for transcutaneous therapies. Each TIZ is accurately focused at a particular depth and heated with smaller pulses (150 ms) to cause shallow zones (1 mm^3^) of coagulation necrosis at a particular site. Nevertheless, the surrounding tissues, as well as the superficial layers, are not affected and persist mostly intact. The low pulse duration limits the occurrence of thermal damage, as also occurs similarly in the laser pulse [46]. The action of MFU causes the following processes: instant contraction of denatured collagen, which activates neocollagen and stimulates thermal stimulation and collagen rebuilding. The consequence of these processes is skin tightening. This is accomplished by setting up minuscule, finely controlled areas of thermal coagulation in the middle and deep dermis, as well as SMAS (superficial musculoaponeurotic system) [41]. The MFU device (Ulthera, Ulthera Inc., Mesa, AZ, USA) is also being used to treat neck, brow, and submentum tissues without using invasive surgical procedures. In these cases, the use of the MFU device (Ulthera, Ulthera Inc., Mesa, AZ, USA) has been approved by the FDA (Food and Drug Administration). There is only one study evaluating the effects of vaginal heating by HIFU (SVELTIA Feminine Manufactured in the City of Cordoba, Argentina) with a vaginal handpiece, including 4 MHz with 3 mm and 4.5 mm-depth vaginal transducers in a group of patients with GSM, SUI, and POP [47]. The authors of this study have demonstrated the efficacy of MFU vaginal application on a histological and clinical level.

Extrapolating the mechanism of action and the results from a MFU study on the skin and a recent study using a vaginal handpiece, we decided to asses influence of MFU Ultravera Hironic on the vaginal wall in women with vaginal laxity (VL) and genitourinary symptoms of menopause (GSM). Taking into account the average thickness of the vaginal wall—3 mm—and its anatomy [48], MFU, with two handpieces—1.5 mm and 3 mm—is capable of affecting the whole thickness of the vaginal wall, which is not possible using lasers and radiofrequency. The 4.5 mm handpiece can act even further, beyond the vaginal wall, affecting adjacent organs, but its deeper action aiming beyond the vaginal wall was not our goal in the presented trial.

## 2. Materials and Methods

### 2.1. Participation in the Study

A total of 20 women with VL and VVA, aged between 48 and 62 (mean age 55.7 years) participated in this nonrandomized, prospective study. The study was approved by the Research Ethics Committee under number 08/KB/VI/2017, and was conducted according to the guidelines recommended by the 2000 Declaration of Helsinki, updated in 2008.

The inclusion criteria included the following: patients with symptoms of GSM and VL, peri-menopause, menopause, and cervical cytology testing negative for cancer. The exclusion criteria included the following: the use of hormone therapy (either systemic or topical) or long-acting moisturizers within 6 weeks prior to the initial assessment, patients with active or recurrent genital infection (e.g., genital herpes, candidiasis, BV), patients with human immunodeficiency virus, recurrent urinary tract infections, pelvic radiation therapy or brachytherapy, reconstructive pelvic surgery or previous vaginal treatment with energy based devices, hyaluronic acid fillers, autologous therapy with plasma derived products (PRP, PRF), or autologous fat transfer.

After history collection and physical examination, patients were selected and instructed about the procedure and after providing information about the aim of the study and informed consent, the patients answered the following questionnaires: FSFI and VLQ. The vaginal status was examined with the use of VHI.

### 2.2. Instruments and Procedures

The MFU therapy course consisted of two treatment sessions, with an interval of 6 weeks, using the MFU device Ultravera, by Hironic. Each treatment session involved the vaginal application of two specially designed probes, respectively 1.5 mm and 3 mm, so as to precisely affect two depths of the vaginal wall. The energy was adjusted based on the patient’s feedback.

The MFU Ultravera device enables all parameters to be selected by software— intravaginal probe rotation: 0–360°; angle: 3–20°; treatment line length: 5–25 mm; pitch: (distance between exposure zones) 1–5 mm; output power: 0.2–2.0 J. Designed for vaginal application, the transducer delivers a series of precise ultrasound pulses along a linear path. For each series of exposures, the following source conditions can be varied: power output, exposure time, length of exposure line, and distance between exposure zones. In this manner, thermal injury can be produced in selective zones, in a straight line at a given depth, in the tissue (a 25-mm line of discrete lesions spaced 0.5–2 mm apart).

Two 360° treatments were performed, starting at a depth of the vaginal canal about 6 cm, according to the insertion mark on the application device, and a second one following the same rule, 3 cm deep in the outer proximal part of the vagina, according to the vaginal probe marking. The procedure started at the 12 o’clock position and finished at the 12 o’clock position. The treatment lines were 25 mm long, each one with a density of one focal point every 1.5 mm (Pitch 1.5), allowing 17 thermal coagulative zones to be created along each line. The intravaginal rotation angle of the probe between shots was 8 (Angle 8), which produced a total of 45 lines shot after a complete rotation in the vagina (360° round). This permitted a gridlike distribution of thermal coagulative zones, with closer spacing along each exposure line than between the parallel exposure lines. The protocol used 2 joules in each shot session, and was sometimes decreased, depending on individual patient’s tolerance.

The questionnaires used in the study were: FSFI and VLQ. The vaginal status was examined with the use of VHI.

## 3. Results

### 3.1. Outcome Measures and Statistic Evaluation

All 20 patients completed the study. All treatment sessions were performed in accordance with the treatment protocol. No adverse events or side effects were observed.

#### 3.1.1. Vaginal Laxity

**Figure 1:**
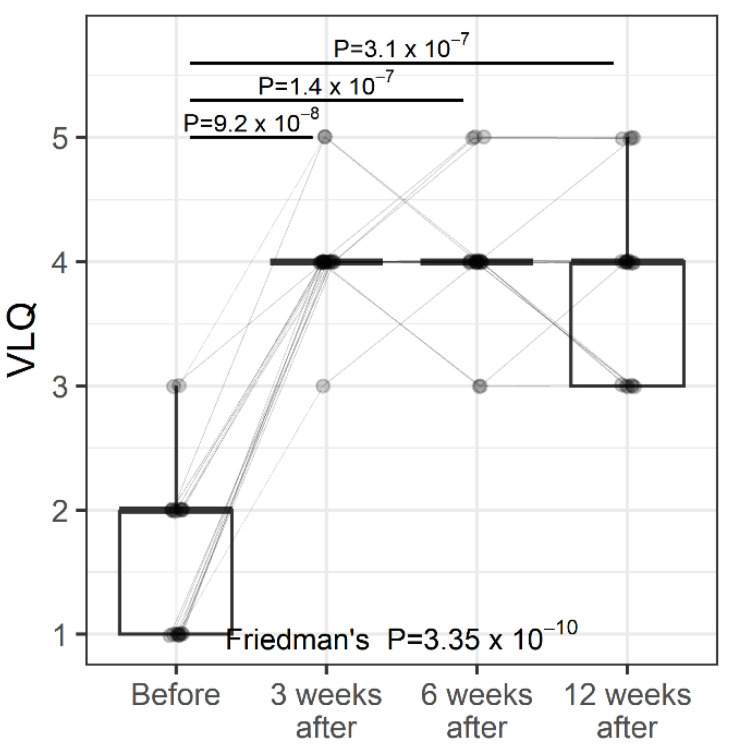
VLQ (Vaginal Laxity Questionnaire) at study entry and follow-up visits (individual values and summary statistics).

Vaginal laxity was assessed by a non-standardized subjective vulvovaginal laxity questionnaire (VLQ) using a 7-point Likert scale. Participants were eligible if they self-reported a perception of vaginal laxity defined as ‘‘very loose’’, ‘‘moderately loose’’, or ‘‘slightly loose’’, on a 7-point Likert scale [16,17]. Data were collected before the first treatment and during a consecutive follow-up visit. Average improvement was calculated. VL changed significantly over time (Figure 1, *p* < 0.0001). Post-hoc comparisons revealed that significant differences were found between study entry and follow-up visits, but not between follow-up visits. The percentage change between the first two weeks (before versus 2 weeks after) ranged from 33.3% to 300%, with a median percentage change of 125%. A total of 8 women showed a change of 100%, one women of 150%, one women of 200%, and eight women of 300%.

Individual values are represented by points joined by lines. Summary statistics are shown using box plots. The middle line of the box plot is the median, and the lower and upper hinges correspond to the first and third quartiles (the 25th and 75th percentiles). The upper whisker extends from the hinge to the largest value (no further than 1.5*IQR from the hinge; IQR is the inter-quartile range—the distance between the first and third quartiles). The lower whisker extends from the hinge to the smallest value (no further than 1.5*IQR of the hinge). The median line overlaps with the lower and upper hinges. The *p*-values were determined using Friedman’s test, followed by a pairwise post-hoc Wilcoxon test with FDR adjustments for multiple testing correction.

#### 3.1.2. Female Sexual Function Index (FSFI)

**Figure 2:**
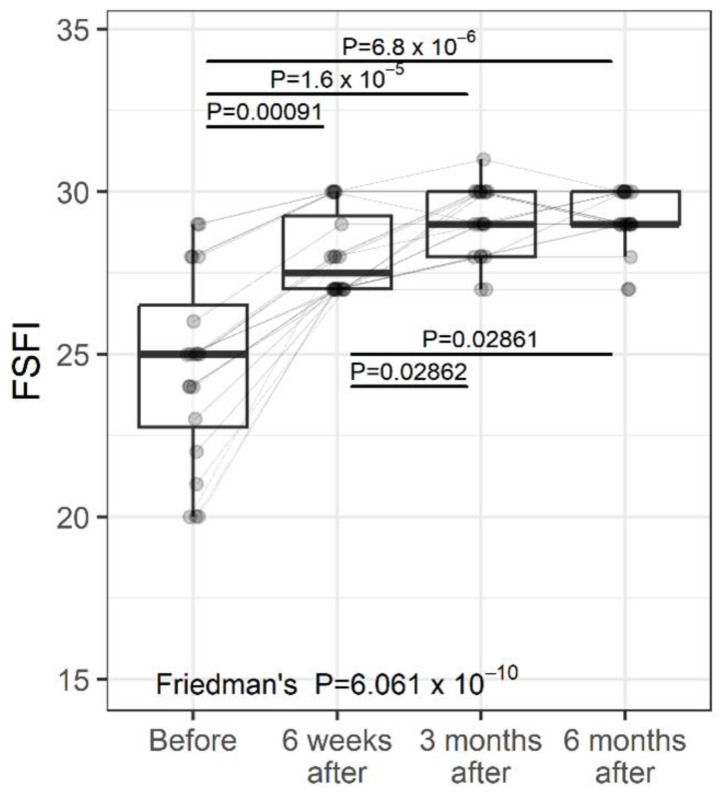
FSFI (Female Sexual Function Index) at study entry and follow-up visits (individual values and summary statistics).

The FSFI is a widely accepted, global evaluation used in female sexual medical trials [49]. The FSFI is a 19-item questionnaire divided into 6 domains: desire, arousal, lubrication, orgasm, satisfaction, and pain, which evaluate a woman’s recent state of sexual function (within the past 4 weeks). The domain scores combine to create a total score (range 2–36). A total FSFI score of 26 is recognized in the medical literature as indicating female sexual dysfunction (FSD). A change in FSFI during the study was significant (Figure 2, *p* < 0.0001). a 6-week follow-up demonstrated significantly improved FSFI scores (from 25 (3.8) to 32 (2.25), median (IQR), *p* < 0.0001) and remained significantly better at 3 months follow-up (29 (2), *p* < 0.0001 versus baseline, and 6 months follow-up (29(1)), *p* < 0.0001 versus baseline. Percentage change—Before vs. 6 weeks: median 12%; 75% of women had a percentage change of at least 7.8%; 25% of women had a percentage change of at least 18.7%. Percentage change—Before vs. 3 months: median 17%; 75% of women had a percentage change of at least 10.4%; 25% of women had a percentage change of at least 25.6%. Percentage change—Before vs. 6 months: median 16.7%; 75% of women had a percentage change of at least 11.2%; 25% of women had a percentage change of at least 25.6%. Percentage change—6 weeks vs. 3 months: median 3.7%; 25% of women had a percentage change of at least 7.1%. Percentage change—6 weeks vs. 6 months: median 3.6%; 25% of women had a percentage change of at least 7.4%.

Individual values are represented by points joined by lines. Summary statistics are shown using boxplots. The middle line of the boxplot is the median; the lower and upper hinges correspond to the first and third quartiles (the 25th and 75th percentiles). The upper whisker extends from the hinge to the largest value (no further than 1.5*IQR from the hinge; IQR is the inter-quartile range—distance between the first and third quartiles). The lower whisker extends from the hinge to the smallest value (no further than 1.5*IQR of the hinge). The *p*-values were determined using Friedman’s test, followed by a pairwise post-hoc Wilcoxon test, with FDR adjustments for multiple testing correction.

#### 3.1.3. Vaginal Health Index (VHI)

**Figure 3:**
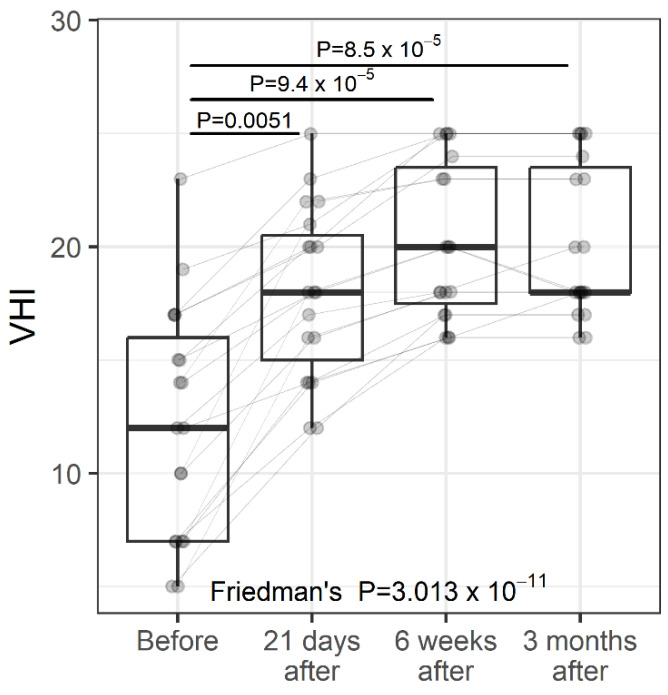
VHI (Vaginal Health Index) at study entry and follow-up visits (individual values and summary statistics).

Vaginal health regarding GSM was evaluated by the Vaginal Health Index (VHI). The VHI consists of the clinical analysis during the specular examination of five parameters and is graded from 1 to 5. The sum of the values of the parameters evaluated results in the total vaginal health score. VHI scores are for vaginal moisture, vaginal fluid volume, vaginal elasticity, pH, and vaginal epithelial integrity. The lower the score, the higher the atrophy. The sum of the values of the evaluated parameters results in the total vaginal health score. When the overall score is less than 15, the vaginal mucosa is considered atrophic [50,51]. VHI was measured at baseline and at three follow-up visits (Figure 3). Overall, there was a significant improvement in the VHI score over time, with the greatest and most significant change occurring between the baseline and at 21 days follow-up (*p* = 0.006). The VHI score leveled off at 21-days follow-up, and no significant changes were found between follow-up visits thereafter. Percentage change—Before vs. 21 days: median 41.7%; 75% of women had a percentage change of at least 18.8%; 25% of women had a percentage change of at least 100%. Percentage change—Before vs. 6 weeks: median 50%; 75% of women had a percentage change of at least 41.4%; 25% of women had a percentage change of at least 128%. Percentage change—Before vs. 3 months: median 64.3%; 75% of women had a percentage change of at least 37.3%; 25% of women had a percentage change of at least 129%.

Individual values are represented by points joined by lines. Summary statistics are shown using boxplots. The middle line of the boxplot is the median; the lower and upper hinges correspond to the first and third quartiles (the 25th and 75th percentiles). The upper whisker extends from the hinge to the largest value (no further than 1.5*IQR from the hinge; IQR is the inter-quartile range—distance between the first and third quartiles). The lower whisker extends from the hinge to the smallest value (no further than 1.5*IQR of the hinge). The *p*-values were determined using Friedman’s test, followed by a pairwise post-hoc Wilcoxon test, with FDR adjustments for multiple testing correction.

## 4. Discussion

Over the past decade, there has been an increase in energy based devices for the treatment of vaginal laxity and genitourinary syndrome of menopause (GSM). Recently, the use of radiofrequency and lasers for the treatment of vaginal laxity and genitourinary syndrome of menopause (GSM) have shown promising treatment outcomes [21,22,23,24,25,26,27,28,29]. In spite of this fact, the FDA, ACOG, and IUGA have launched a critical position, which has been questioned by the group of renowned experts [28]. Little is known about the effects of the acoustical energy of micro-focused ultrasound in the vaginal wall and its influence on vaginal laxity and related to menopause vaginal atrophy. Contrary to lasers and RF that concentrate their energy superficially, affecting the vaginal epithelium and lamina propria, with no deeper action, the acoustical energy of micro-focused ultrasound can penetrate further, affecting the whole vaginal thickness, with no detrimental effect on adjacent organs. However there is a kind of terminological confusion concerning acoustical energy based devices operating on a basis of focused ultrasound, and a clear distinction should be made between HIFU and MFU, depending on energy and action in the tissue what was described in detail in the introduction. With MFU devices, which have been used in aesthetic medicine, there is no cavitation and ablation of tissue; the action relies only on thermal effect and the resulting heat-dependent contraction of denatured collagen and the induction of neocollagenesis. The action of MFU in tissue and its clinical effects in aesthetic medicine is well established and documented [41,42,43,44,46], but there is a scarcity of evidence regarding its effects on the vaginal wall. There is only one study in the existing literature describing the influence of an acoustical energy-based device operating on the basis of focused ultrasound in the management of vaginal atrophy, vaginal hyperlaxity, and stress urinary incontinence. The authors first presented the effectiveness of HIFU vaginal application with 3 mm and 4.5 mm handpieces, at a histological and clinical level [47]. Histological changes included: epithelium and lamina propria thickening, better vascularization of the lamina propria, and an increase in estrogens receptor expression in the vaginal wall. Statistically significant clinical improvement has been noted for GSM, SUI (stress urinary incontinence), and POP (pelvic organ prolapse) and has been evaluated using generally approved tools: FSFI (Female Sexual Function Index), VHI (Vaginal Health Index), ISIQ-SF (International Consultation on Incontinence Questionnaire-Short Form), and PISQ-12 (Pelvic Organ Prolapse/Urinary Incontinence Sexual Function Questionnaire). In a described study, a term HIFU was been used; however, given the energy, frequency, and depth of the action in the vaginal wall, the term MFU would be better suited to this method.

In our study we used the MFU device Ultravera, by Hironic. Although Ultravera has three vaginal handpieces—1.5 mm, 3 mm, and 4.5 mm—in our study, we used only the 1.5 mm and 3 mm devices, given that the average thickness of the vaginal wall is 3 mm [48]. In our opinion, a 4.5 mm handpiece is capable of affecting tissue adjacent to the vagina and could possibly influence the urethra and SUI, as was described by the authors of the study cited above. In our trial, we focused on the influence of MFU on GSM and VL, and this is why we did not use the 4.5 mm handpiece, which enables action beyond the vaginal wall. Our protocol of treatment and the results evaluated by FSFI, VLQ, and VHI concerning GSM and VL have been consistent with the studies cited above, and they provide evidence of the effectiveness and safety of MFU in a vaginal application.

Our trial has several limitations: (1) the lack of a control arm and an open-label design, (2) the small sample size, and (3) a relatively short follow-up duration. Despite the limitations, MFU therapy performed twice in the vaginal canal showed promising efficacy and safety profiles. This has motivated us to start another study to assess the histological changes of the vaginal wall by means of biopsies, as well as by evaluating the concentrations of collagen and elastin before and after treatment.

## Data Availability

The data presented in this study are available on request from author, P.K., upon reasonable request.
